# Dynamic Analysis of Closed Die Electromagnetic Sheet Metal Forming to Predict Deformation and Failure of AA6061-T6 Alloy Using a Fully Coupled Finite Element Model

**DOI:** 10.3390/ma15227997

**Published:** 2022-11-12

**Authors:** Zarak Khan, Mushtaq Khan, Se-Jin Yook, Ashfaq Khan, Muhammad Younas, Muhammad Zeeshan Zahir, Muhammad Asad

**Affiliations:** 1 Department of Mechanical Engineering, HITEC University, Taxila 47080, Pakistan; 2 Mechanical Engineering Department, Prince Mohammad Bin Fahd University, Al-Khobar 31952, Saudi Arabia; 3 School of Mechanical Engineering, Hanyang University, Seoul 04763, Korea; 4 School of Engineering, Robert Gordon University, Aberdeen AB10 7GJ, UK; 5 Department of Mechanical Engineering, University of Engineering and Technology, Peshawar 25000, Pakistan

**Keywords:** electromagnetic forming, Lorentz force, deformation, dynamic analysis

## Abstract

This research presents a fully coupled 3D numerical model to analyse the dynamics of high-speed electromagnetic forming process for aluminium alloy AA6061-T6. The effect of Lorentz force distribution, velocity and kinetic energy on deformation, the bounce back effect and failure of the sheet has been investigated. Experiments were performed for AA6061-T6 alloy using an 18.750 KJ electromagnetic forming machine for varying the sheet thickness (0.5 mm, 1.02 mm and 1.63 mm) compared with the simulation results. The results showed that increasing the sheet thickness increases the Lorentz force due to a higher induced current. The inertial forces were more pronounced in thicker sheets (1.63 mm) as compared to the thinner sheets (0.5 mm and 1.02 mm), resulting in a higher bounce back effect for the thicker sheet. The numerical model accurately predicted the sheet failure for the 0.5-mm sheet, as also observed from the experimentation. The sheet deformation from simulations was found to be in good agreement with the experimental results.

## 1. Introduction

Sheet metal forming is one of the most widely used manufacturing process in the industry, with applications ranging from automotive to aerospace industries. The formability of sheets in the conventional sheet metal-forming process depends on a number of factors, such as material properties, blank holding force, material flow during forming and die design, to mention a few [1]. However, the major limitations include low formability, uneven thickness variations due to a nonuniform distribution of forces, high spring-back and higher wrinkling of the material [2]. The electromagnetic forming process has the advantage of contact-free application of force, environmental friendly, better process control, reduced rework, reduced tooling cost, reduced spring-back and improved formability [2].

In the electromagnetic forming process, the workpiece material achieves forming velocity ranging between 100 m/s and 300 m/s [3]. The workpiece deforms due to transient magnetic pressure. At very high speed, the formability of a deforming workpiece increases while the spring-back effect minimizes [2]. Due to the complexity of the electromagnetic forming process involving multiple process parameters and its effects on sheet deformation such as magnetic pressure (Lorentz force), electrical conductivity of the material and behaviour of the material under high strain rate, many researchers have developed numerical models to investigate these effects. Takatsu et al. [4] used a spiral coil for free bulging of the aluminium workpiece and validated the experimental results with a numerical model. Nonuniform distribution of radial magnetic force was observed, which led to uneven deformation of the blank in free bulging. The Lorentz forces acted only on the annular region, while the central region of the workpiece deformed due to inertial force. Fenton and Daehn developed a 2D Arbitrary Langrangian–Eulerian (ALE) model to analyse the magnetic force distribution and sheet morphology during deformation. The model evaluated the magnetic force on every time step and the corresponding motion of the workpiece during deformation. Due to complexity, the model was limited to 2D free bulging. Oliveria et al. [5] used a loose coupling model to investigate the deformation of the workpiece material during magnetic forming, and commercial software LS DYNA was used. The magnetic pressure was first estimated and then applied to the workpiece. ABAQUS/Explicit commercial code was used by Correia et al. [6] to estimate the maximum deformation of the workpiece. The model was uncoupled, hence easy to develop and converge, but relatively exaggerated Lorentz force results were approximated that resulted in the overestimation of sheet deformation as compared to experimental deformation results. A sequentially coupled model was developed by Haiping et al. [7] for magnetic pulse forming of thin tubes as the workpiece. In the model, the change in the magnetic field due to the moving workpiece was ignored. The model was good for a 2D axisymmetric workpiece but not suitable for complex geometries or unsymmetric shapes. The adaptive remeshing technique was used by Cui et al. [8] in a loosely coupled model to remesh the air domain in the surroundings. The model gave better results for 2D axisymmetric deformation as compared to experimental results. Another uncoupled numerical model was developed by Li et al. [9] using ANSYS/EMAG to evaluate the magnetic pressure on the workpiece for all time steps, and the calculated magnetic pressure was then applied on the workpiece using ABAQUS/Explicit software for 3D deformation. The results obtained were satisfactory with reduced computational time as compared to previous studies. Cao et al. [10] developed a fully coupled numerical model using COMSOL Multiphysics to validate the experimental results of Takatsu et al. [4] for free-forming axisymmetric sheet deformation. The change in the induced current due to changing the sheet morphology was also considered. The results obtained were very accurate; however, the model is time-consuming and is difficult to implement on 3D models. Yu et al. [11] analysed circular hole flanging using the conventional method and electromagnetic forming process. The results were compared, and much better formability was observed in the electromagnetic forming process due to consistent radial force and inertial forces on the workpiece as compared to the conventional method in which localised elongation occurred. Noh et al. [12] developed an uncoupled model for unsymmetric 3D magnetic forming of the aluminium alloy. The results obtained were good but with some errors. A sequentially coupled numerical model was adopted to analyse the input parameters of pulsed forming. The effect of the changing morphology of the workpiece on Lorentz’s force was not considered. The results were satisfactory [13]. A loosely coupled numerical model was developed by [14] to analyse the deformation of the corrugated and ribbed workpieces. The ribbed sheet showed better deformation and magnetic pressure due to the higher value of the skin depth. Huang et al. [15] developed a pulsed magnetic forming setup to control the magnetic pressure and blank holding force. Edge wrinkling phenomena were reduced in the final workpiece, and the formability was increased. Ning Lui et al. [16] varied the coil parameters to analyse the Lorentz force distribution on the workpiece by changing the diameter of the spiral coil. It was observed that, by changing the coil parameters, the Lorentz force distribution and workpiece velocity can be altered, and consequently, its final deformed shape may vary accordingly. Zarak Khan et al. [17] analysed three important process parameters for closed die aluminium alloy forming. It was observed that the most important process parameters in electromagnetic sheet metal forming are the input voltage and workpiece thickness. The coil parameters play smaller roles as compared to voltage and sheet thickness. Ductile failure is a common phenomenon in the sheet metal-forming process due to excessive tensile stresses, resulting in failure of the workpiece [18]. Considerable effort has been made to predict the ductile failure of various materials using a modelling approach. A combination of a forming-limiting diagram, ductile fracture criterion and shear stress criterion has been used to predict the fracture strain of steel and aluminium alloys [19]; however, a generalised model has not been developed for different materials. In the current research, the von Mises yielding criterion is used incorporated with the failure index [20] to predict failure during electromagnetic sheet metal forming.

In the current research, a 3D fully coupled numerical model was developed to investigate the dynamic behaviour of electromagnetic sheet forming to predict the deformation and failure. Electrical circuits coupled with magnetic field and solid mechanics were used to model the electromagnetic forming process. Experimentation was performed on aluminium alloy AA6061-T6 of varying sheet thicknesses. The sheet failure and deformation predicted by the numerical model were validated using experimentation.

## 2. Equivalent Circuit

**Figure 1:**
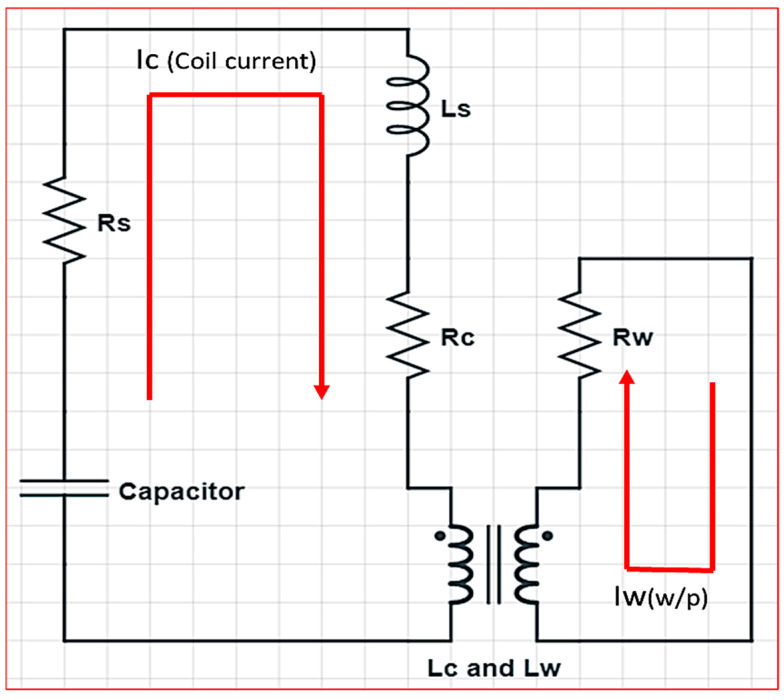
RLC Equivalent circuit of the EMF process.

The electromagnetic forming circuit consists of the overall system inductance and resistance represented by L_s_ and R_s_, respectively. Other important components of the circuit are workpiece resistance (R_w_), inductance (L_w_) of the workpiece, coil resistance (R_c_), coil inductance (L_c_), and mutual inductance (M) between the coil and workpiece, as shown in Figure 1. The mathematical relation of the important parameters of the electric circuit has been formulated by Mamalis et al. [21].

(1) $$L=L_{s}+L_{c}-\frac{M^{2}}{L_{w}}$$

(2) $$R=R_{s}+R_{c}+\frac{M^{2}}{{L_{w}}^{2}\;}R_{w}$$

(3) $$M=K\sqrt{L_{c}L_{w}}$$

In Equation (3), K is the coupling factor between the metal workpiece and copper coil. The transient current can be calculated by using the governing Equation (4). The important parameters in calculating the current are presented in Table 1 as follows [22].

(4) $$I\left(t\right)=\frac{U0}{\omega L\;}e^{-\beta t}\sin\left(\omega t\right)$$

(5) $$\beta =\frac{R}{2L}$$

(6) $$\omega =\sqrt{\frac{1}{\mathrm{LC}}-\beta^{2}}$$

## 3. Numerical Modelling

A fully coupled model was established consisting of three main modules, as discussed below.

### 3.1. Electrical Circuit

As discussed in the previous section, the important variables are the inductance of the system (L_s_), the resistance of the system (R_s_), capacitance (C) and voltage (U_0_). The designed numerical model works on transient current Equation (4) to calculate the time-dependant current.

### 3.2. Magnetic Module

The magnetic field variables are calculated using the following equations [22].

(7) $$\nabla \times \vec{H}=\vec{J}$$

(8) $$\nabla \times \vec{E}=\frac{-d\vec{B}}{dt}$$

(9) $$\nabla \times \vec{B}=0$$

(10) $$\vec{J}=\frac{\vec{I_{c}}}{s}=\sigma_{e\;}\vec{E}$$

Equations (7)–(10) are differential forms of Maxwell’s equations. Equation (7) represents Maxwell’s fourth equation, also known as modified Ampere’s circuital law. Equation (8) represents Faraday’s law of electromagnetic induction. Equation (9) represents Gauss’s law of magnetism. The important parameters in calculating the magnetic field are presented in Table 2.

### 3.3. Solid Mechanics Module

Due to the transient current in the coil, the induced current in the workpiece is produced that results in the Lorentz force between the coil and workpiece. The Lorentz force produced deforms the workpiece based on displacement equilibrium [22] Equation (11).

(11) $$\rho \frac{d^{2}\bar{u}}{{\mathrm{dt}}^{2}}-\Delta \sigma s=\bar{\mathrm{fm}}$$

where ρ is the density, $\bar{u}$ is the displacement vector, σs is stress tensor and $\bar{\mathrm{fm}}$ is the electromagnetic force density. As electromagnetic forming is a high-speed deformation process, the high strain rate effect on the mechanical properties of the workpiece must be defined. Generally, there are three models majorly used for high-speed deformation: (a) the Steinberg model [23] of Equation (12) (simplified for AA6061-T6 with an initial yield strength of 93 MPa), (b) the Johnson–Cook model [24] of Equation (13) and (c) the Cowper–Symonds model [25] of Equation (14), respectively.

(12) $$\sigma =93{\left(1+125\varepsilon \right)}^{0.1}$$

(13) $$\sigma =\left[A+B{\left(\varepsilon \right)}^{n}\right][1+\mathrm{Cln}(\dot{\varepsilon })]$$

(14) $$\bar{\sigma }=\sigma_{y}\left[1+{\left(\frac{\dot{\bar{\varepsilon }}}{p}\right)}^{m}\right]$$

The Cowper–Symonds model uses fewer material properties as compared to the Johnson–Cook. The Johnson–Cook model is limited in flow stress compared to Cowper–Symonds model at higher strain rates [26]. Therefore, based on the above, the Cowper-Symonds model was selected for this research. The results obtained from the Cowper–Symonds model were observed to be in close approximation to the experimental results [17].

**Figure 2:**
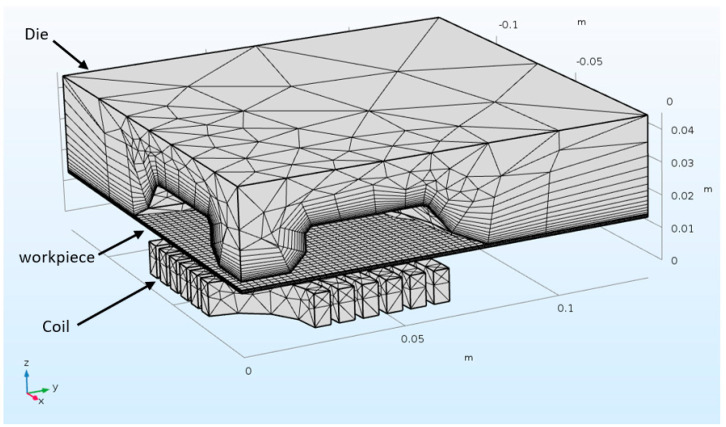
Mesh of EMF closed die for the electromagnetic forming of AA6061-T6.

Table 3 shows the electrical and mechanical properties of the sheet and coil used in the simulation and experimentation. The mesh of the model is shown in Figure 2. A fully coupled 3D quarter model was developed to simulate the electromagnetic forming process. The coil and air domains were included in the electromagnetic module and were excluded in the solid mechanics module. In the solid mechanics module, the die and blank holder were rigid to reduce the simulation time. The die domain was boundary meshed, and the workpiece was mapped meshed, while the remaining geometry was tetrahedrally meshed. The mesh contains 121,635 domain elements, 14,101 boundary elements and 1134 edge elements. The electrical and mechanical properties of the workpiece and coil, along with Cowper–Symonds constants used in the numerical model, are presented in Table 3. After convergence tests, a mesh of 121,635 elements was established, resulting in a processing time of 280 min.

### 3.4. Numerical Model Flow Chart

**Figure 3:**
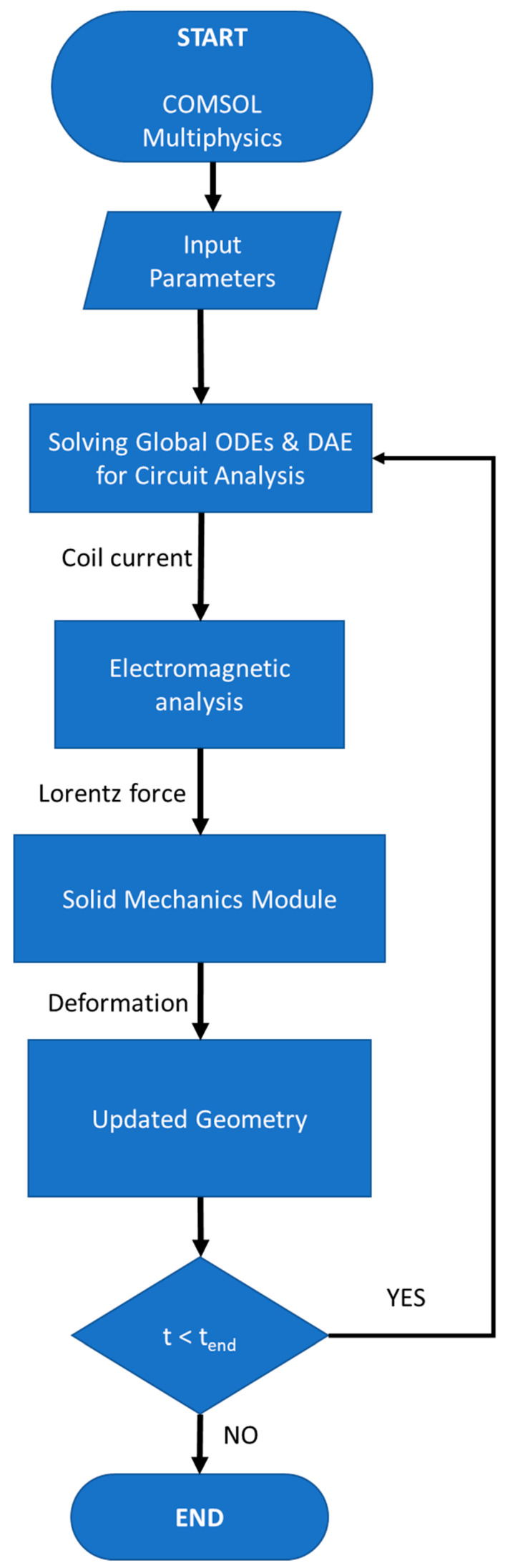
Flow chart of the finite element method.

The flow chart shown in Figure 3 represents the numerical model used in the research. The transient current was calculated using a circuit analysis by solving Equation (4) in COMSOL. The current was then passed through the coil to generate an induced magnetic field. Equations (7)–(10) were used to calculate the current density and magnetic flux. For Lorentz force calculation, Equation (11) was used. The magnetic force generated was used as an input load in solid mechanics to deform the workpiece. After the deformation at each time step, the cycle is repeated; hence, the effect of change in the geometry on the inductance of the system is also considered. Once the Lorentz force and inertial force diminish, the numerical analysis ends.

## 4. Experimental Setup

**Figure 4:**
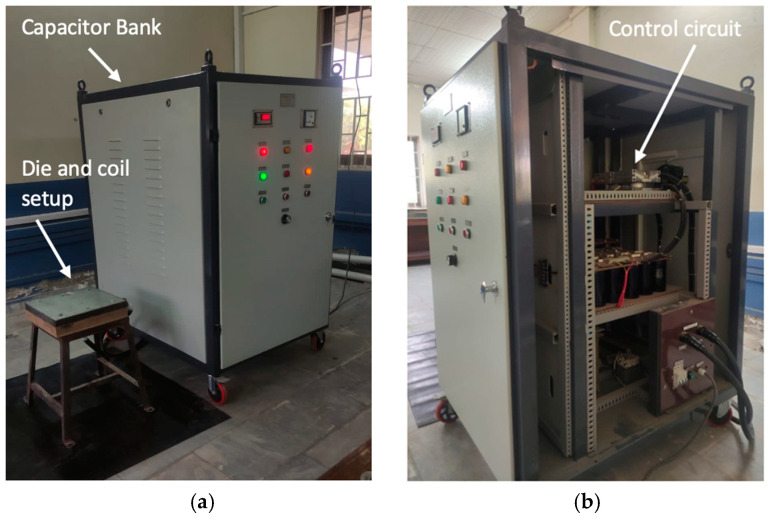
(**a**) Electromagnetic forming setup. (**b**) Internal configuration of the capacitor bank with control circuit and capacitors.

To validate them, the numerical model experiments were performed on three different parameters. The experimental setup consisted of a 6 × 10^−3^ F capacitor bank, power supply ranging from 400 V to 3000 V and the inductance and system resistance were 3.63 × 10^−6^ H and 0.02 Ω, respectively (Figure 4). Three AA6061-T6 sheets with varying thicknesses of 0.5 mm, 1.02 mm and 1.63 mm, respectively (SWG 25, 18 and 16), were deformed at a constant input energy of 18.750 KJ. The selected range of sheet thickness is very important in the automotive industry, specifically in door panels, roofs, support flanges, etc. [27].

### 4.1. Coils Preparation

**Figure 5:**
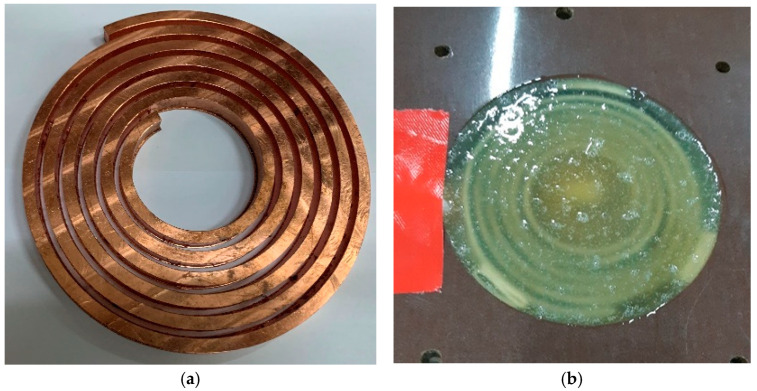
(**a**) Copper coil. (**b**) Coil gap covered in epoxy.

The tool coil was machined out of 10-mm copper plate using 3-Axes CNC Milling (MV-1060 YDPM, Taiwan) with an automatic 24 tool changer. The coil was covered with epoxy resin for reinforcement, as shown in Figure 5. The dimensions and resistivity of the coil are given in Table 4.

### 4.2. Die Machining

**Figure 6:**
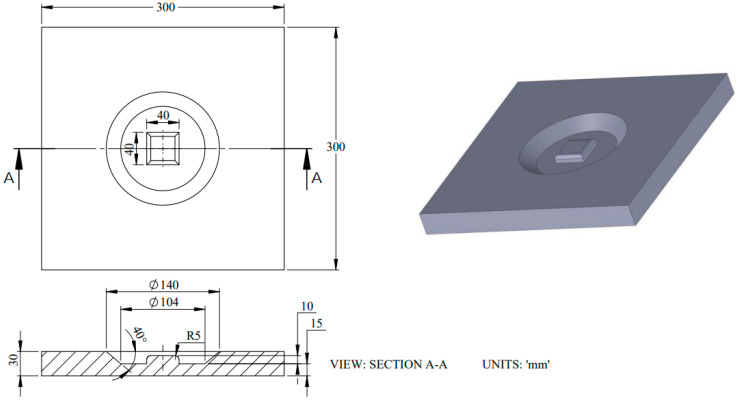
Three-dimensional CAD model and drawing for the die used in electromagnetic forming experimentation.

**Figure 7:**
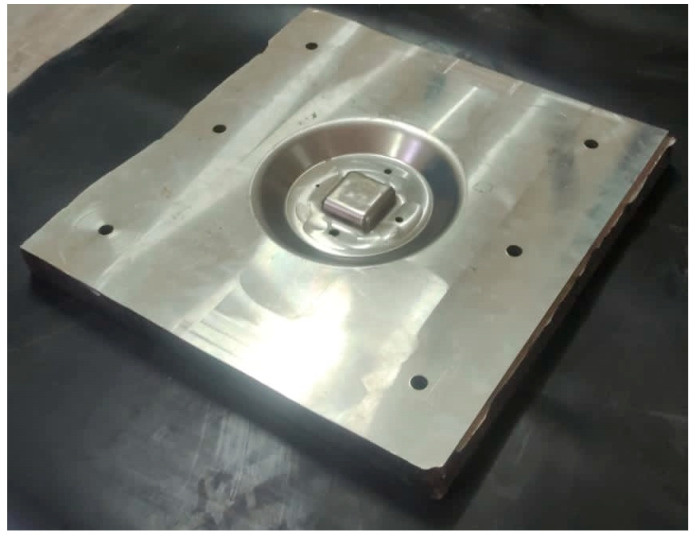
Die for experimentation.

A CNC milling machine was used to machine the die from 300 mm × 300 mm × 40 mm SS304 steel alloy (Figure 6). A middle block of 40 mm × 40 mm with small air vents of 3 mm were also machined and drilled, respectively. The air vents are used to evacuate the entrapped air between the workpiece and die, as shown in Figure 7. The workpiece thickness range, according to the Standard Wire Gauge (SWG), was 25, 19 and 16 (0.5 mm, 1.02 mm and 1.63 mm, respectively).

## 5. Results and Discussion

### 5.1. Lorentz Force Distribution

Even though the Lorentz force increases with the thickness of the sheet, the increase in Lorentz force due to increased skin depth is very small compared to the plastic strain energy required to deform the thicker sheet; therefore, the total deformation and plastic strain energy obtained reduce for a thicker sheet. It can also be observed that the effective Lorentz force duration is from 50 microseconds to 150 microseconds, which is a very short time compared to the deformation time. The major deformation occurs due to inertial forces, but the initial impulsive Lorentz force plays important role in generating these inertial forces, which is why it is important to calculate the Lorentz forces for analysing and designing the electromagnetic forming process.

**Figure 8:**
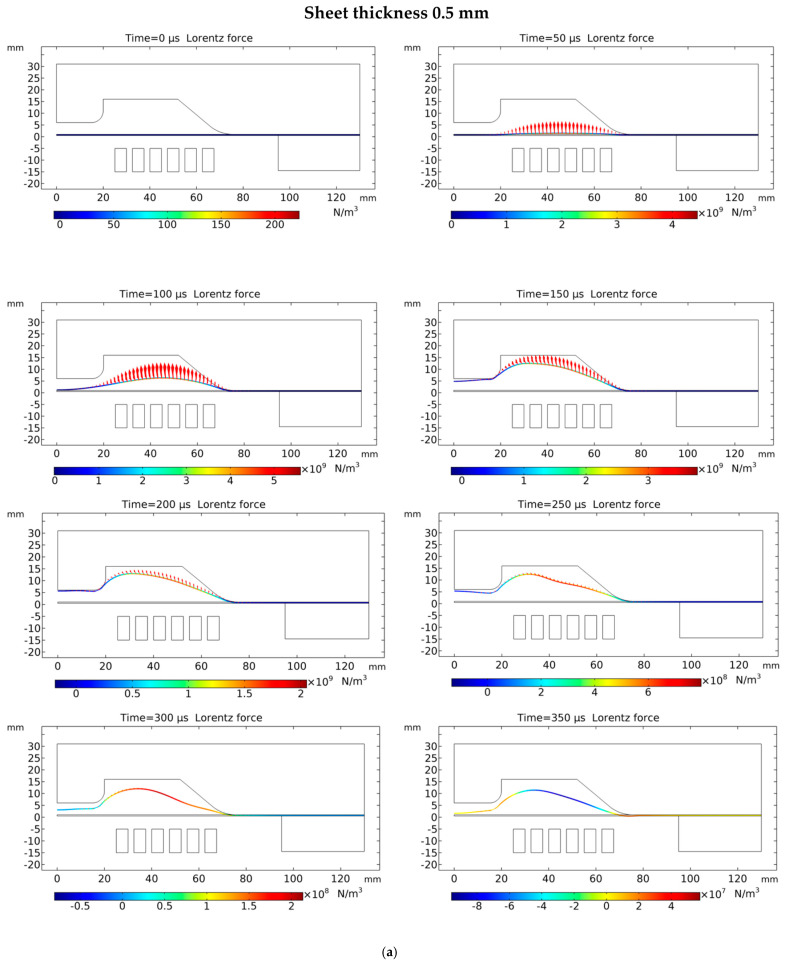

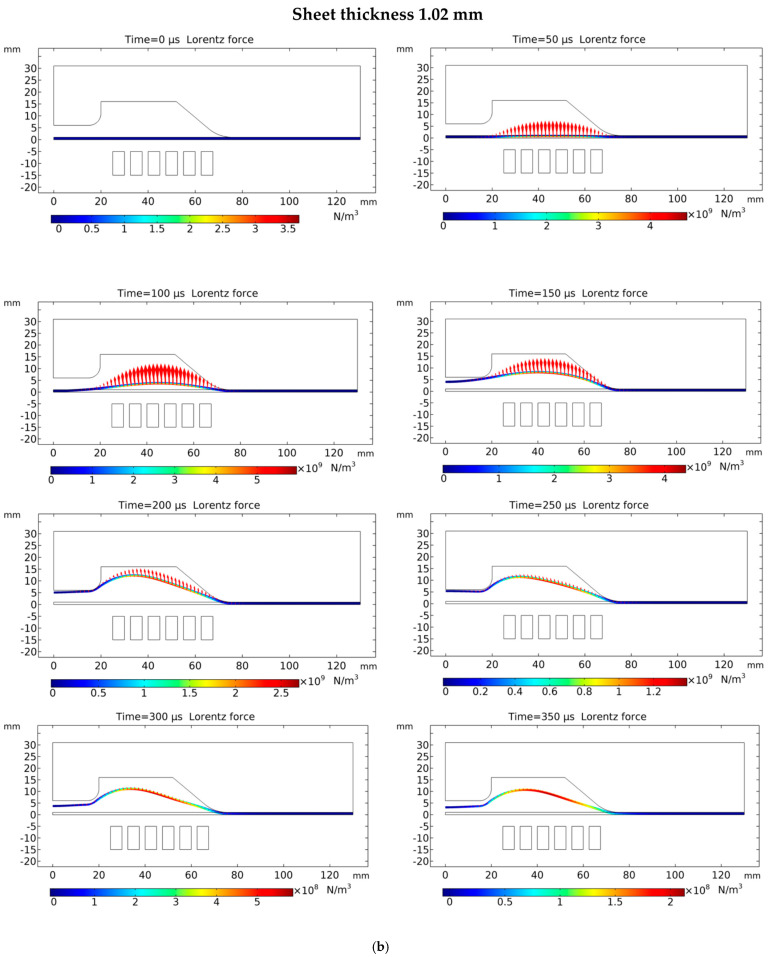

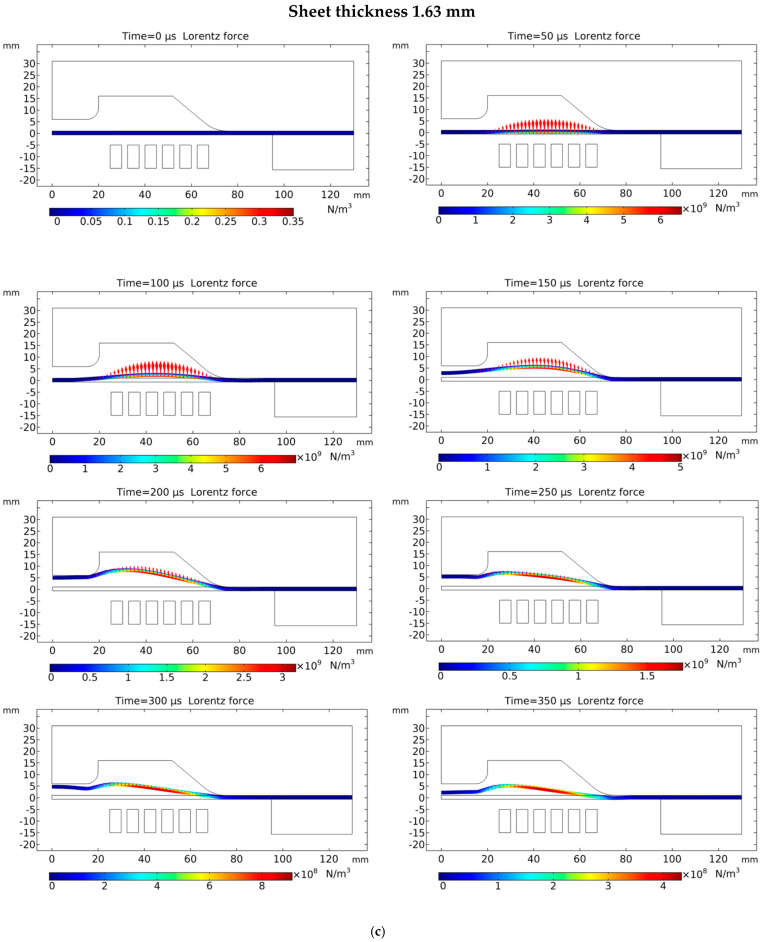
Lorentz force distribution at different time steps: (**a**) 0.5-mm sheet, (**b**) 1.02-mm sheet and (**c**) 1.63-mm sheet.

The results obtained from the 3D numerical analysis are shown in this section. The Lorentz force distribution on the workpiece affects the geometry of the sheet. Figure 8 represents the force distribution on the sheets at all conditions at several time steps. From Figure 8, it is observed that the Lorentz forces mainly act on the area of the sheet that is right on top of the coil. The force distribution of the 0.5-mm sheet has the lowest value of 5.7 × 10^9^ N/m^3^ at the time step at 80 μs. Lorentz force on the 1.02-mm sheet has a maximum value of 6.2 × 10^9^ N/m^3^ at 85 μ, while that on the 1.63-mm sheet is 7.149 × 10^9^ N/m^3^ at time step 90 μs. The reason for the shift in time and magnitude of maximum forces on varying sheet thicknesses can be explained by the work of Dordizadeh et al. [28]. The magnitude of the Lorentz force depends on the induced current and magnetic flux; due to varying sheet thicknesses, the induced current varies, which results in varying magnetic pressures. The induced current for thicker sheets has a higher value because of the larger cross-sectional area [29]. Additionally, there are some losses in the form of attractive forces in thicker sheets due to phase shifting between the induced current and eddy current. This shift occurs because, at different depths, the current changes its waveform to penetrate the deeper layers. The Lorentz force on the thicker sheet is the highest, yet its deformation height is lowest; the reason for that is a thicker sheet having more mechanical strength is difficult to deform [28]. From the numerical results of all three conditions, it is observed that the Lorentz force diminishes quickly, and the remaining deformation occurs under the inertial force. This phenomenon was also claimed by Kleiner et al. [30].

### 5.2. Velocity of Sheet

**Figure 9:**
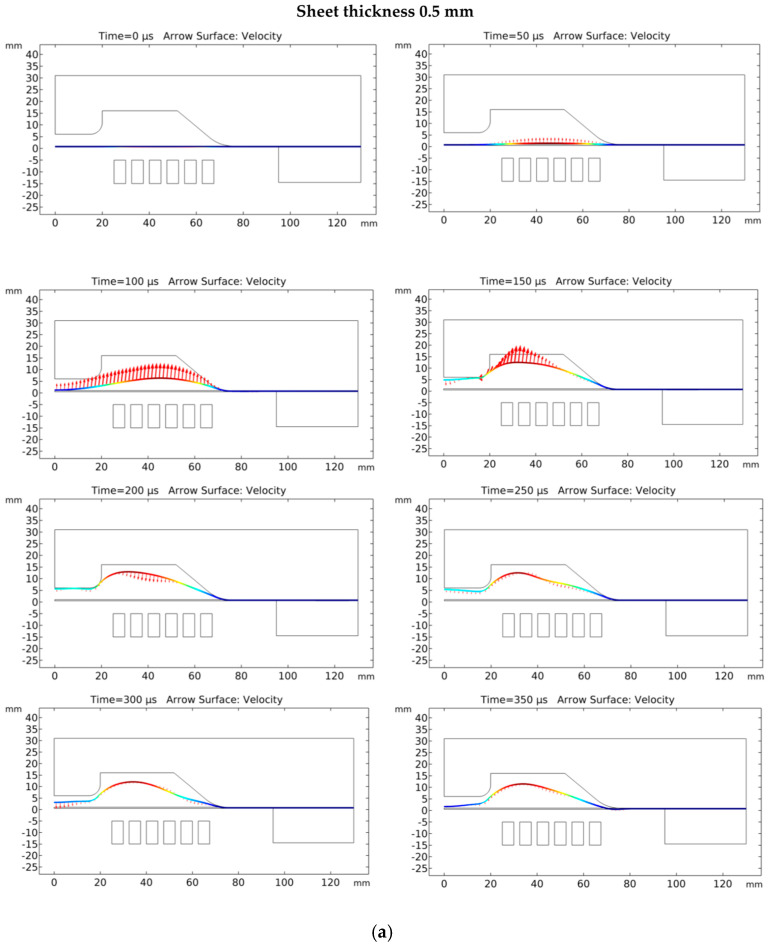

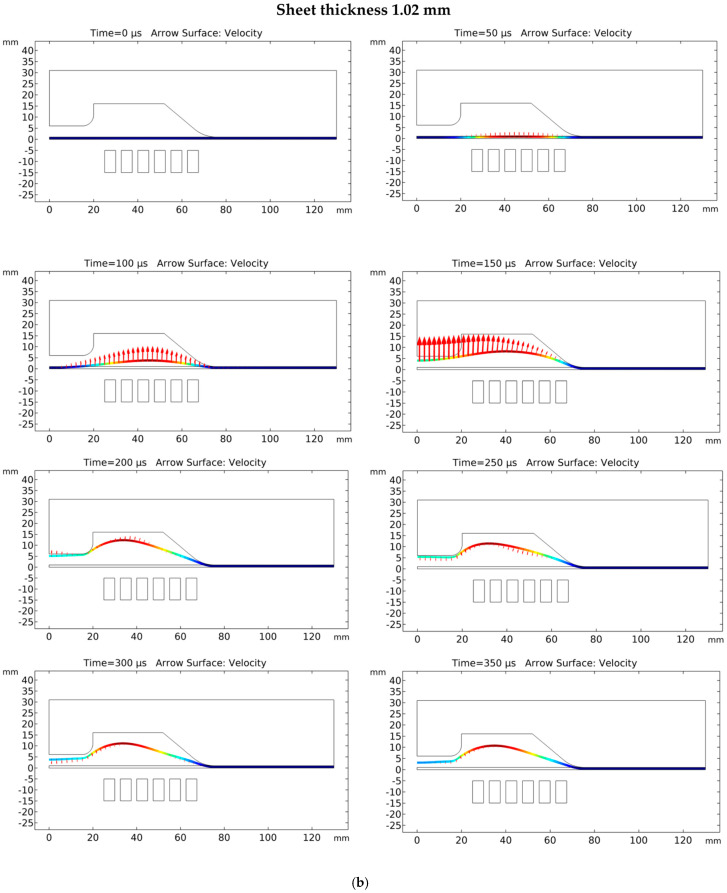

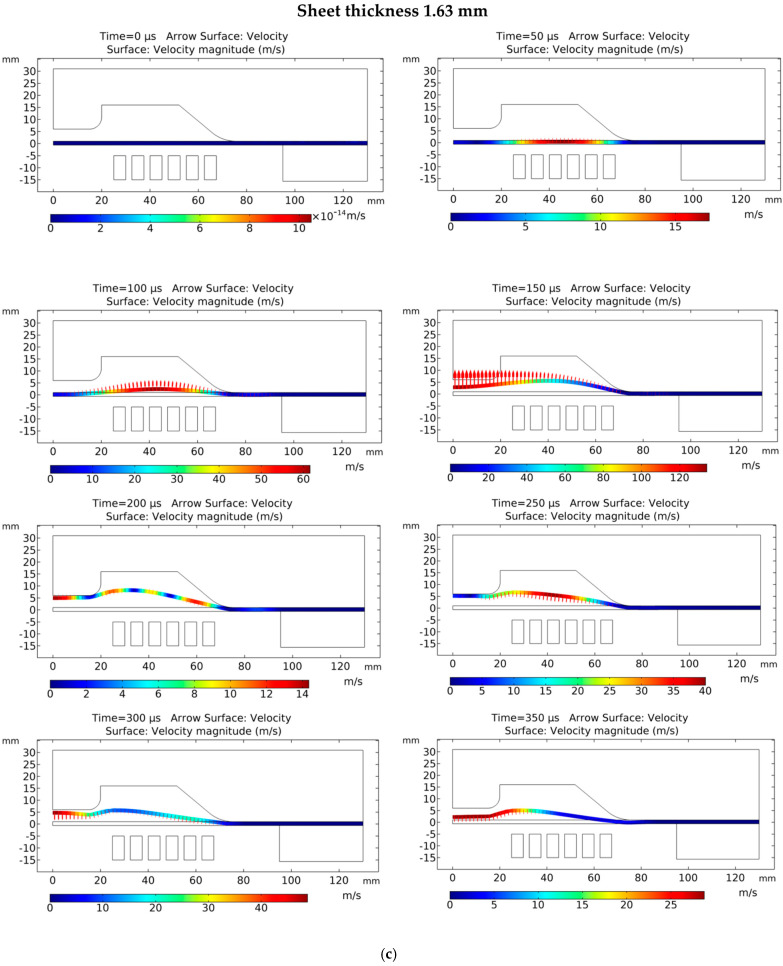
Distribution of the axial velocity of the workpiece at different forming stages: (**a**) 0.5-mm sheet, (**b**) 1.02-mm sheet and (**c**) 1.63-mm sheet.

The velocity of the deforming workpiece is dependent on the magnitude of Lorentz’s force. The workpiece deforms under the influence of kinetic energy when Lorentz’s force diminishes [16]. Therefore, it is important to study the velocity distribution on the deforming workpiece. The comparison of velocity distribution at all conditions at several time steps is shown in Figure 9. The 0.5-mm sheet achieved the highest velocity of 200 m/s at time step at 135 μs. The maximum velocity attained by the sheet of 1.02-mm thickness was 180 m/s at time step 150 μs. The velocity of the 1.63-mm-thick sheet was the lowest among the three 160 m/s at time step 165 μs due to its greater mechanical strength [28]. After 200 μs, the velocity changes its direction and reduces significantly because of the sheet and die interaction. The bounce-back velocity of the 0.5-mm sheet at 200 μs was the highest with a 60-m/s magnitude. The 1.02-mm sheet attained a 50-m/s velocity, while the 1.63-mm sheet had a minimum magnitude of 20 m/s. The deformation of the workpiece after the Lorentz force diminishes mainly occurs due to kinetic energy provided to the workpiece by pulsed magnetic pressure. Due to different velocity distributions, the final morphology of the workpiece at all three conditions is also different.

### 5.3. Kinetic Energy

**Figure 10:**
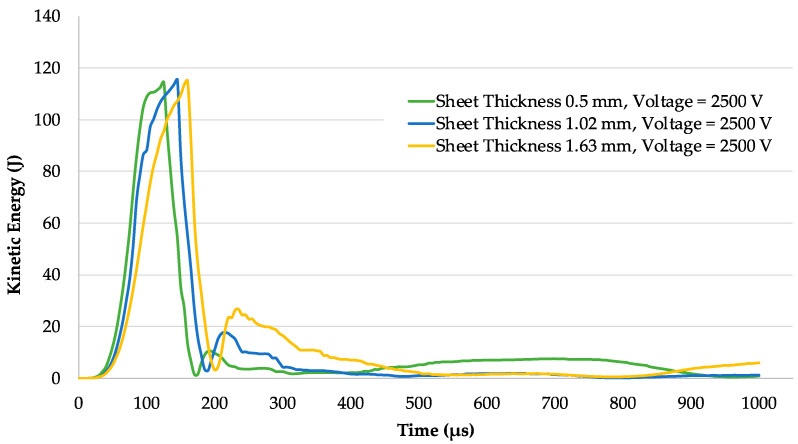
Kinetic energy of sheets with different thicknesses.

In the electromagnetic forming process, the workpiece achieves its kinetic energy from the initially applied Lorentz force. The kinetic energy then transforms into plastic strain energy during the inertial deformation of the workpiece. In such high-speed forming, the inertial effect plays a vital role during the deformation of the workpiece. The plastic strain energy is achieved at the expense of the kinetic energy to attain the final shape during deformation after the Lorentz force diminishes [16]. The kinetic energy that converts into plastic strain energy and eventually deforms the workpiece is dependent on the velocity achieved by the workpiece during the initial impulse provided by the Lorentz forces. Therefore, it is important to study and analyse the velocity and Lorentz force distribution on the workpiece during high-speed electromagnetic forming [16]. The contribution of inertia is above 55% in the case of an open die or round simple die [16]. The inertial component in a closed die with the central block or another complicated shape die will be less as compared to an open die because of the workpiece and die interaction. The reason is kinetic energy abruptly reduces after the collision of the workpiece in a closed die. From Figure 10, the peak kinetic energy of the 0.5-mm sheet is 118 J at 115 μs. The kinetic energy then abruptly reduces to 0 at 170 μs because of the collision of the sheet with the die surface. After that, another peak of 8 J can be seen at 200 μs, which then diminishes at nearly 300 μs. The sheet with 1.02-mm thickness shows the same 118 J peak at 155 μs. The kinetic energy then abruptly reduces to 0 at 190 μs because of the collision of the sheet with the die surface. After that, another peak of 19 J can be seen at 200 μs, which then diminishes at nearly 400 μs. The sheet with 1.63-mm thickness shows the same 118 J peak at 170 μs. The kinetic energy then abruptly reduces to 0 at 205 μs because of the collision of the sheet with the die surface. After that, another peak of 30 J can be seen at 220 μs, which then diminishes at nearly 505 μs. The shift in the curves is due to the inertial difference, which is mainly due to the difference in mass of the sheets. Overall, the kinetic energy of a thick sheet will be higher as compared to thinner sheet sizes. The difference is mainly due to the difference in mass of the sheets and the increase in induced current in the thick sheet [29].

### 5.4. Sheet Failure

**Figure 11:**
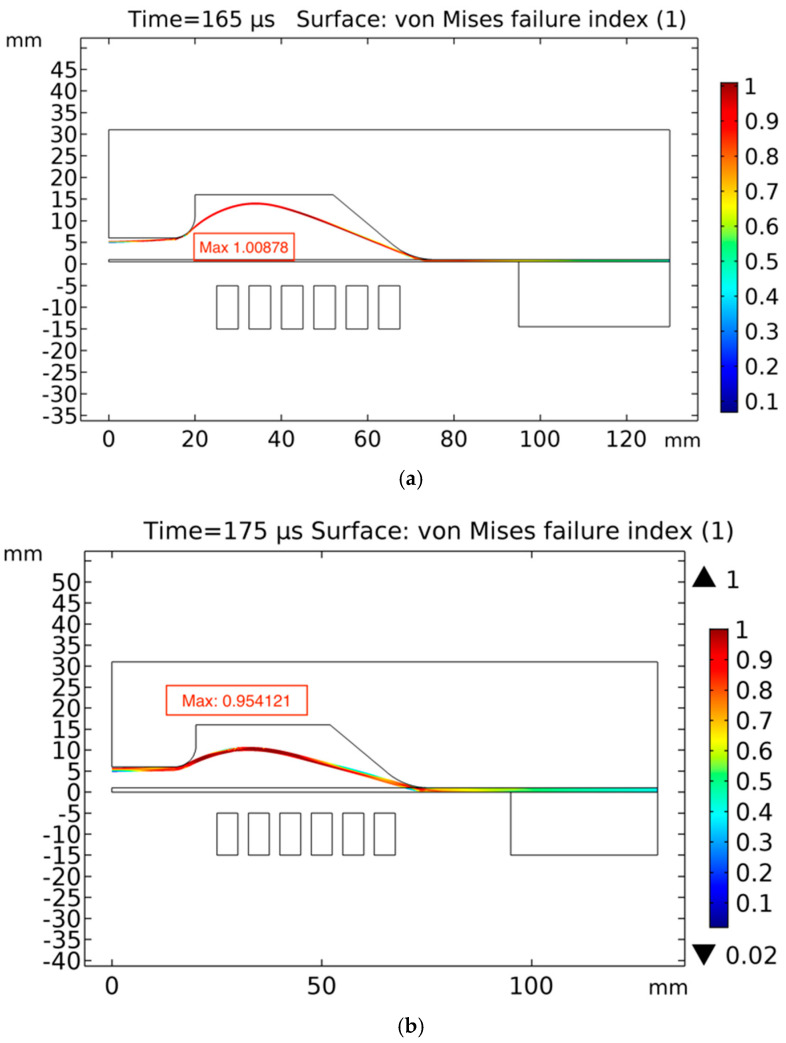
von Mises failure index of sheets at 18.75 KJ: (**a**) 0.5 mm and (**b**) 1.02 mm.

**Figure 12:**
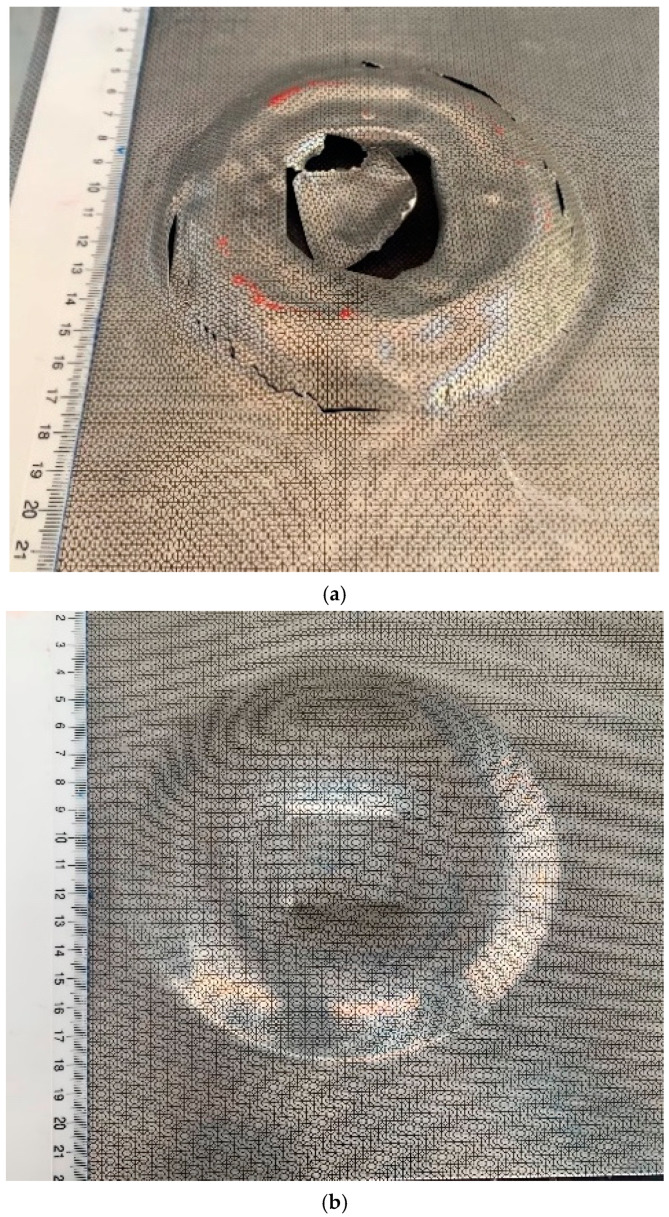
Experimentally deformed sheets at 18.750 KJ energy: (**a**) 0.5 mm sheet and (**b**) 1.02 mm sheet.

The sheets were evaluated for the estimation of failure using the von Mises failure index. The model presented here is not a fracture mechanics model and cannot be used to predict the exact fracture location, fracture mechanics or crack propagation, which will be incorporated in the future; however, it is an estimation of the ductile deformation of the material. The von Mises failure index is the ratio between the computed flow stress and the given limit, which, in this case, is the experimental results measured by [31]. A failure index equal to or greater than ‘1′ indicates failure of the sheet material. Values lower than ‘1′ lie in the safe zone [20]. The ultimate tensile strength of high-speed forming AA6061-T6 was experimentally measured by A. Manes [31] for failure prediction, and the maximum value was 570 MPa. Figure 11a shows the failure index of the 0.5-mm sheet. The maximum value was 1.00878 at 165 μs, which exceeded ‘1′; hence, according to the failure index, the sheet would fail. From Figure 11b, it can be observed that the 1.02-mm sheet reached its maximum failure index at 175 μs. The maximum value was 0.954, which lies in the safe zone. The corresponding results were verified from the experimental results, as shown in Figure 12a–c.

A Forming Limit Diagram (FLD) for AA6061-T6 at high strain rates was developed by Woo et al. [32] based on the Marciniak–Kuczynski theory (M–K) theory and high strain rate electrohydraulic forming process. It was observed that the strain rate increases, forming a limit line, i.e., resulting in higher formability of the material. A micro-mechanistic constitutive model was developed by Nguyen et al. [33] using Dung’s porous ductile material model and coupled with the Hill’48 quadratic yield function to predict the forming limiting curves using the M–K theory for the AA6061-T6 alloy. For the quasistatic deep drawing of the AA6061-T6 alloy, Djavanroodi and Derogar [34] developed a forming limiting curve to predict safe and failure zones in deep drawn sheets. Based on the literature, it can be concluded that high strain rates delay the necking and fracture of the material, resulting in high formability as compared to the quasistatic sheet forming process.

**Figure 13:**
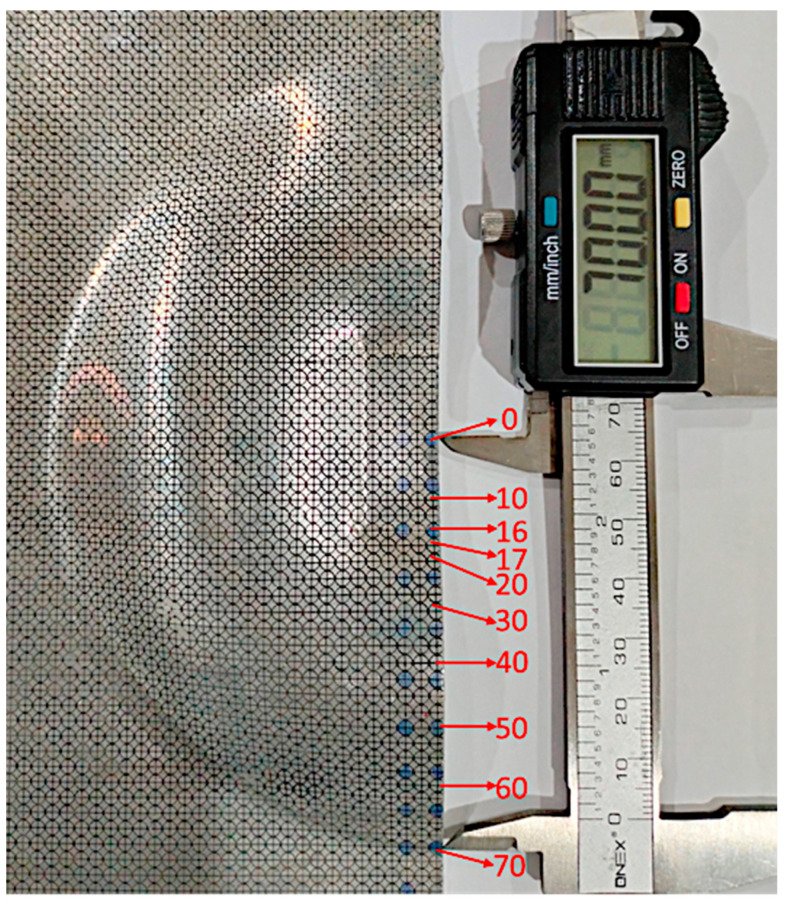
Measurement point locations on the deformed AA6061 T6 sheet.

**Figure 14:**
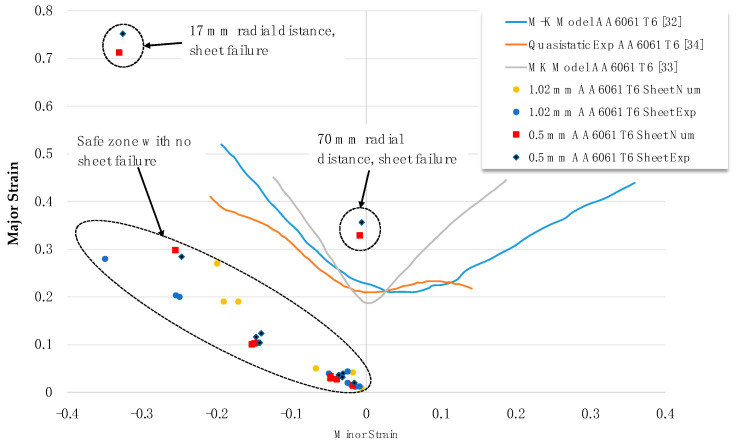
Simulation and experimental major and minor strain for 1.02-mm and 0.5-mm sheets for AA6061 T6 alloy.

The experimental and simulated results of major and minor strain for 0.5-mm and 1.02-mm sheets at 10 different points (Figure 13) measured using a circle grid analysis is presented in Figure 14, along with the quasistatic FLD and high strain rate FLD from the literature. The experimental values of the strain were measured five times, and the average values are presented in Figure 14. Table 5 presents the measured strain values for the closed die electromagnetic forming. The FLD from the literature is based on the manufacturing of a dome, whereas the results from the current model are based on the closed die forming. Therefore, a direct comparison of the total strain from the closed die forming with the dome cannot be performed; however, the high strain associated with the sheet failure can be compared. It is observed that the major and minor strains predicted numerically were in good agreement with the experimental results. From Figure 14, it is evident that all the points marked on the 1.02-mm sheet lie in the safe forming zone, with few points in the insufficient stretch zone. However, in the 0.5-mm sheet, most of the points lie in the safe forming zone, except for two points at 17-mm and 70-mm radial distances from the centre of the sheet, which is above the safe zone (also highlighted in Table 5). From the literature, it is evident that the major strain is safe until 0.35 [35,36]. Although the simulation can estimate the location of possible failure, the model cannot predict the exact location of the fracture of the sheet or direction of the fracture, which highlights the need for a detailed micromechanistic fracture model and future model development.

### 5.5. Effective Plastic Strain

**Figure 15:**
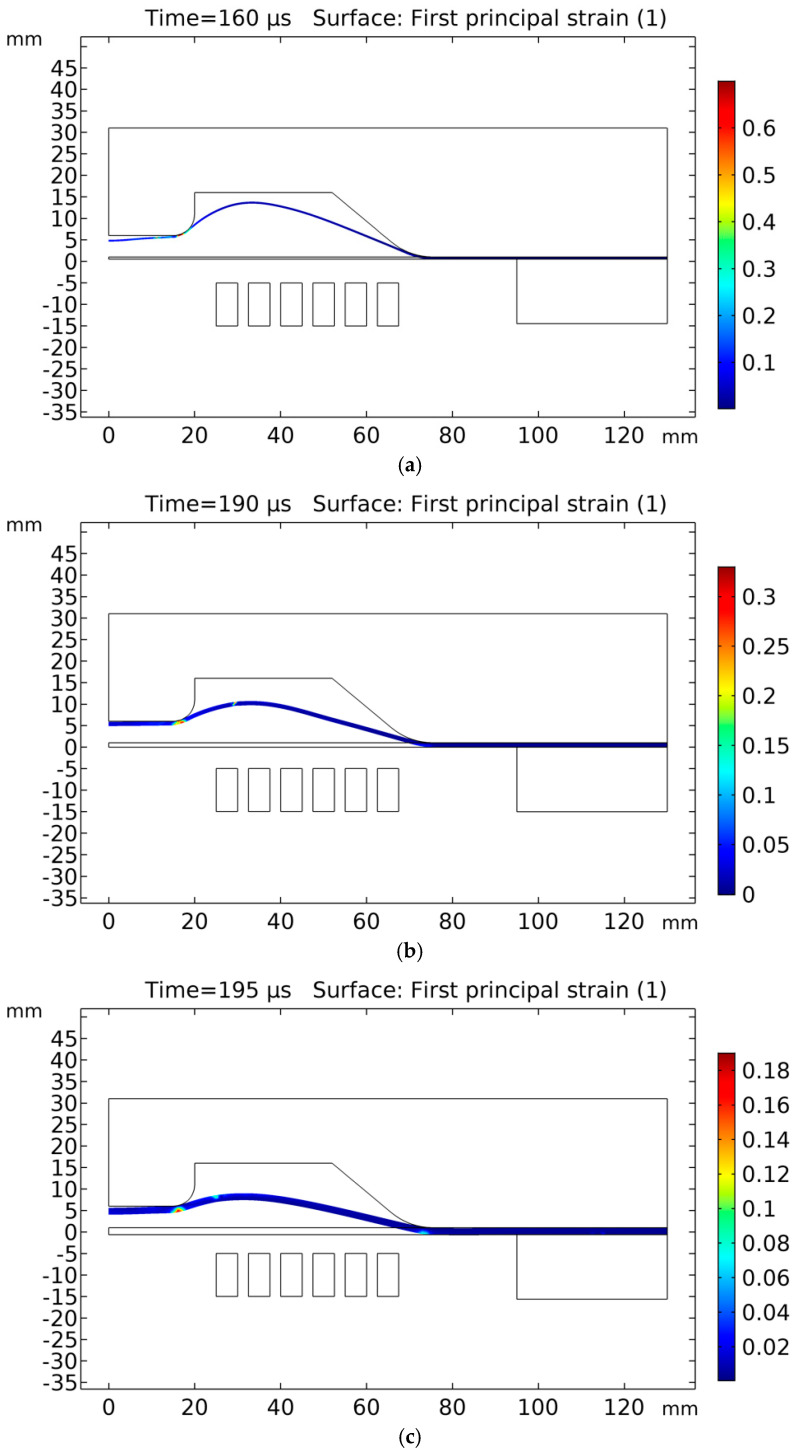
Effective plastic strain: (**a**) 0.5-mm sheet, (**b**) 1.02-mm sheet and (**c**) 1.63-mm sheet.

The effective plastic strain of the sheets was numerically estimated and presented in Figure 15. The effective plastic strain of the 0.5-mm sheet was 70% at 145 μs, as shown in Figure 15a. The maximum effective plastic strain of the 1.02-mm sheet was 31% at 180 μs, as shown in Figure 15b, and the maximum effective plastic strain of the 1.63-mm sheet was 18% at 195 μs, as shown in Figure 15c. From the results, it can be concluded that, even though the Lorentz force on a thicker sheet is the highest still, the effective plastic strain in the thinner sheets will be higher. A thicker sheet has a high rigidity and will need greater force to deform. The maximum strain in the electroforming of aluminium, as evident from the work of [35], can reach up to 35%. The forming limiting diagram of AA6061-T6 at a high strain rate also confirms the failure of the 0.5-mm sheet, in which the plastic strain reached up to 70%, which is very high from the estimated limits [35]. The effective plastic strain in repulsive magnetic forming is higher towards the centre of the workpiece [37]. The effective plastic strain is highest near the central region of the workpiece, where the die geometry changes abruptly; the thickness of the sheet has the lowest values at that region, as discussed by [17]; furthermore, the velocity of the sheet is also highest at the same region, which reveals the effect of effective plastic strain on the forming of the sheet. In general, the higher the effective plastic strain, the better formability due to inertial forces.

## 6. Comparison of Experimental and Numerical Deformation

**Figure 16:**
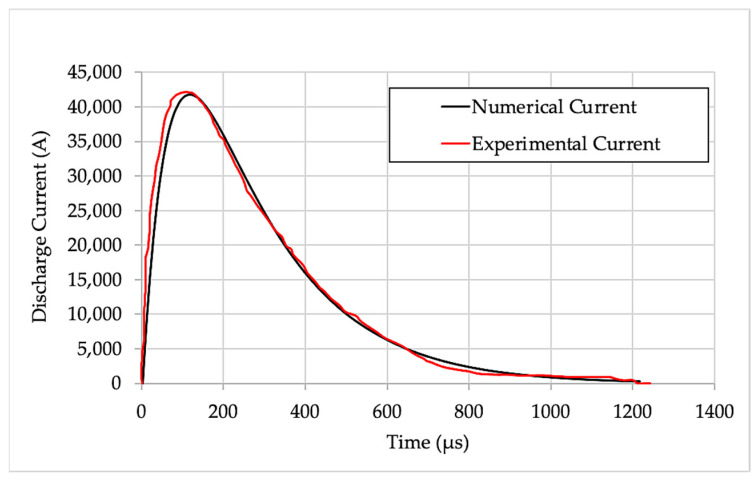
Discharge current at 2500 V.

**Figure 17:**
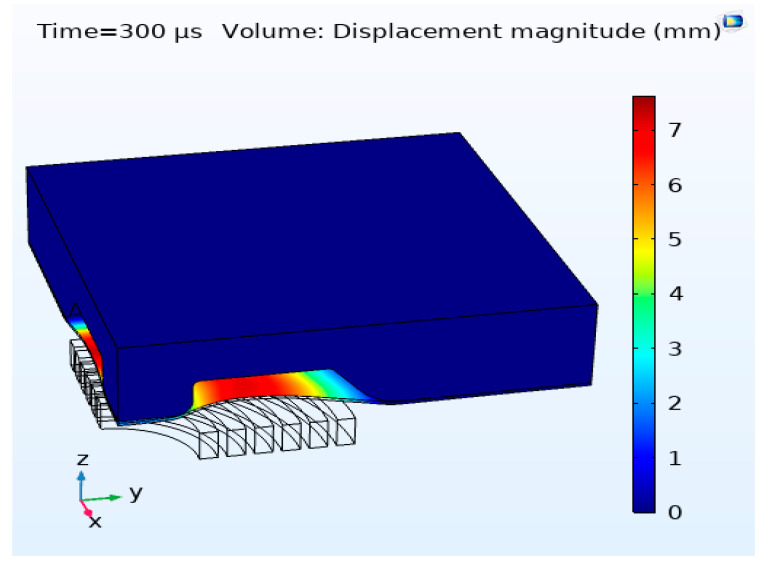
Three-dimensional numerical results for a sheet thickness of 1.63 mm.

**Figure 18:**
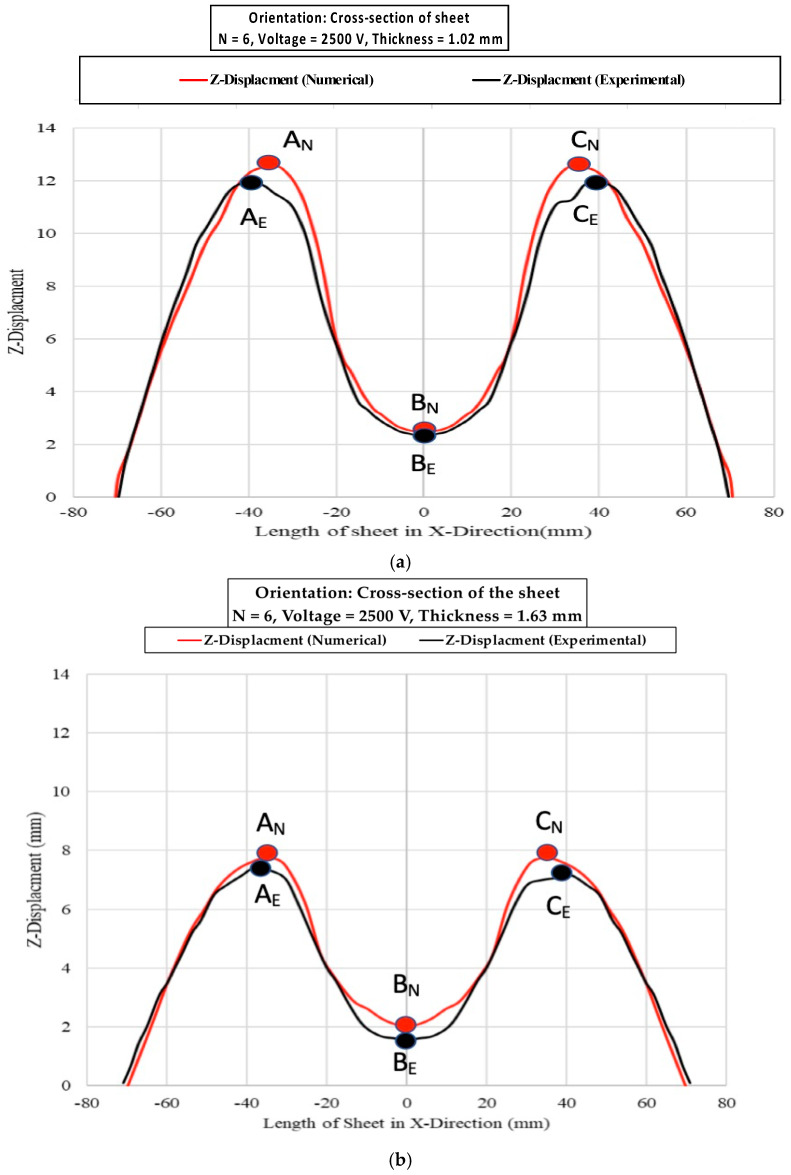
Numerical and experimental results (Z-Displacement): (**a**) 1.02 mm and (**b**) 1.63 mm.

A comparison of the experimental and numerical discharge current is presented in Figure 16. The spiral coil was modelled as six concentric rings, and the internal resistance and inductance of the system were considered in estimating the numerical current curve. The experimental and numerical current curve are in good agreement. The input energy used for all experimental conditions was 18.750 KJ. Figure 17 shows a 3D numerical simulation of 1.63-mm sheet thickness. The experimental deformation in the 1.02-mm and 1.63-mm-thick workpieces was measured using a laser scanner. The cross-section of the deformed profile for both conditions was plotted against numerical results, as shown in Figure 18. The numerical deformation curve was compared with the experimental sheet deformation curve. The curves were compared at six distinct points: A_N_, A_E_, B_N_, B_E_, C_N_ and C_E_, as shown in Figure 18. The deformation heights in the Z-direction are tabulated in Table 6 below with the percentage errors. The numerical results are close to the experimental results; the error is attributed to the personal errors during measurements and the coil and sheet gap during electromagnetic forming [38].

## 7. Conclusions

This research presents a fully coupled 3D numerical model and experimental analysis of sheet deformation and failure in electromagnetic forming of AA6061-T6. A detailed comparison of numerical and experimental results was performed. The Lorentz force distribution was observed to increase with increase in sheet thickness. The 0.5-mm sheet has the lowest Lorentz force value (5.7 × 10^9^ N/m^3^) followed by 1.02-mm sheet (6.2 × 10^9^ N/m^3^), while the maximum for the 1.63-mm sheet was 7.149 × 10^9^ N/m^3^. The maximum velocity was observed for 0.5 mm sheet (200 m/s) as compared to 1.02 mm and 1.63 mm sheets. The rebound velocities of the sheets followed the same trend. This is because the thicker sheet, due to its higher mechanical strength, needs more force and time to start deformation. From the kinetic energy of the three sheets during deformation it was observed that the effect of inertia of thicker sheet was more as compared to the thin sheets, which resulted in increased bounce back effect. The numerical model correctly predicted the von Mises failure index for sheets of various thicknesses as also validated by the experimental results. The 0.5 mm sheet was observed to shear (at 18.75 KJ) in simulation as well as experimentation. Major and minor strains can also be used to estimate the failure of the sheet. The major strains produced in the 0.5 mm sheet was around 0.6 which indicates the chance of failure as in normal circumstance AA6061-T6 alloy will fail after 0.45. The strains in 1.02 mm and 1.63 mm sheets were 0.3 and 0.18 respectively. Overall, the numerical results were in good agreement with experimental results. Future research includes a fully coupled closed die 3D numerical simulation including mesh deletion damage model for damage and failure analysis to further analyse the damage behaviour of various materials. 

Future works will include the development of FLD for electromagnetic forming of AA6061 T6 through experimentation development of the model to predict FLD and a detailed micromodel for fracture mechanics to accurately predict the onset of material failure.

## Figures and Tables

**Table 1:** Important parameters in the current calculations.

Sr	Parameters	Symbols	Values (Units)
1	System Inductance	L_s_	3.63 µH
2	System Resistance	R_s_	0.02 Ω
3	Capacitance	C	0.006 F
4	Damping Coefficient	β	0.75
5	Current Frequency	ω	21,000 rad/s

**Table 2:** Important parameters in the magnetic field module.

Sr	Parameter	Symbol
1	Magnetic intensity	$\overset{\;}{H}$
2	Current density	$\vec{J}$
3	Single turn cross section	s
4	Magnetic flux Density	$\vec{B}:$
5	Electric intensity	$\vec{E}$
6	Electrical conductivity	$\sigma_{e}$

**Table 3:** Electrical and mechanical properties of the sheet and coil (selected values are taken from [8]).

Serial	Component/Material	Properties	Parameter	Values
1	Forming Coil/Copper	Resistivity	ρ	1.72 × 10^−8^ m
		Self-Inductance	L_c_	3.63 µH
		Resistance	R_s_	0.02 Ω
2	Sheet/AA6061-T6	Resistivity	ρ	2.65 × 10^−8^ m
		Poison’s ratio	v	0.35
		Density	ρ	2980 kg/m^3^
		Elastic Modulus	E	69.0 GPa
3	Cowper–Symonds model [8]	Constants	p	$6500\;s^{-1}$
			m	0.25

**Table 4:** Dimensions and resistivity of copper coil.

Outer Diameter	Inner Diameter	Width of Cross Section	Height	Pitch of the Coil	Number of Turns N	Resistivity
140 mm	50 mm	5 mm	10 mm	2.5 mm	6	1.72 × 10^−8^ ohm-m

**Table 5:** Major and minor strain for 1.02-mm and 0.5-mm sheet thicknesses.

	1.02-mm Sheet Thickness				0.5-mm Sheet Thickness			
Radial Distance (mm)	Num Major Strain	Exp Major Strain	Num Minor Strain	Exp Minor Strain	Num Major Strain	Exp Major Strain	Num Minor Strain	Exp Minor Strain
0	0.028	0.020	−0.049	−0.025	0.103	0.104	−0.150	−0.143
10	0.050	0.040	−0.068	−0.050	0.101	0.116	−0.153	−0.148
16	0.190	0.204	−0.171	−0.255	0.299	0.284	−0.256	−0.247
17	0.270	0.280	−0.200	−0.350	0.713	0.804	−0.331	−0.326
20	0.035	0.040	−0.049	−0.050	0.104	0.124	−0.145	−0.141
30	0.190	0.200	−0.191	−0.250	0.034	0.040	−0.048	−0.031
40	0.013	0.016	−0.015	−0.020	0.030	0.032	−0.048	−0.032
50	0.012	0.012	−0.013	−0.015	0.028	0.036	−0.040	−0.037
60	0.009	0.012	−0.006	−0.009	0.014	0.020	−0.018	−0.016
70	0.041	0.044	−0.018	−0.025	0.3289	0.356	−0.009	−0.006

**Table 6:** Numerical and experimental results of the workpiece displacement in the Z-direction.

Sheet Thickness	Displacement in Z-Direction Numerical Model			Displacement in Z-Direction Experimental [mm]			Percentage Error		
	A_N_	B_N_	C_N_	A_E_	B_E_	C_E_	A	B	C
1.02 mm	12.6	2.2	12.8	12	2	12.1	5%	10%	5.785%
1.63 mm	7.6	2	7.6	7.4	1.8	7.1	2.7%	10%	7%

## Data Availability

Not applicable.

## References

[1] Shang J. (2006). Electromagnetically Assisted Sheet Metal Stamping. Ph.D. Thesis.

[2] Psyk V., Risch D., Kinsey B.L., Tekkaya A.E., Kleiner M. (2011). Electromagnetic Forming—A Review. J. Mater. Process. Technol..

[3] Zittel G. A Historical Review of High Speed Metal Forming. Proceedings of the 4th International Conference on High Speed Forming (ICHSF2010) Columbus.

[4] Takatsu N., Kato M., Sato K., Tobe T. (1988). High-Speed Forming of Metal Sheets by Electromagnetic Force. JSME Int. J. Ser. 3 Vib. Control. Eng. Eng. Ind..

[5] Oliveira D.A., Worswick M.J., Finn M., Newman D. (2005). Electromagnetic Forming of Aluminum Alloy Sheet: Free-Form and Cavity Fill Experiments and Model. J. Mater. Process. Technol..

[6] Correia J.P.M., Siddiqui M.A., Ahzi S., Belouettar S., Davies R. (2008). A Simple Model to Simulate Electromagnetic Sheet Free Bulging Process. Int. J. Mech. Sci..

[7] Haiping Y.U., Chunfeng L.I., Jianghua D.E.N.G. (2009). Sequential Coupling Simulation for Electromagnetic-Mechanical Tube Compression by Finite Element Analysis. J. Mater. Process. Technol..

[8] Cui X., Mo J., Li J. Research on Homogeneous Deformation of Electromagnetic Incremental Tube Bulging. Proceedings of the 6th International Conference on High Speed Forming.

[9] Xu J.R., Yu H.P., Li C.F. (2013). Effects of Process Parameters on Electromagnetic Forming of AZ31 Magnesium Alloy Sheets at Room Temperature. Int. J. Adv. Manuf. Technol..

[10] Cao Q., Li L., Lai Z., Zhou Z., Xiong Q., Zhang X., Han X. (2014). Dynamic Analysis of Electromagnetic Sheet Metal Forming Process Using Finite Element Method. Int. J. Adv. Manuf. Technol..

[11] Yu H., Zheng Q., Wang S., Wang Y. (2018). The Deformation Mechanism of Circular Hole Flanging by Magnetic Pulse Forming. J. Mater. Process. Technol..

[12] Noh H.G., Song W.J., Kang B.S., Kim J. (2014). 3-D Numerical Analysis and Design of Electro-Magnetic Forming Process with Middle Block Die. Int. J. Precis. Eng. Manuf..

[13] Mamalis A.G., Manolakos D.E., Kladas A.G., Koumoutsos A.K. (2006). Electromagnetic Forming Tools and Processing Conditions: Numerical Simulation. Mater. Manuf. Process..

[14] Lei X., Tan J., Zhan M., Gao P. (2018). Dependence of Electromagnetic Force on Rib Geometry in the Electromagnetic Forming of Stiffened Panels. Int. J. Adv. Manuf. Technol..

[15] Huang Y., Lai Z., Cao Q., Han X., Liu N., Li X., Chen M., Li L. (2019). Controllable Pulsed Electromagnetic Blank Holder Method for Electromagnetic Sheet Metal Forming. Int. J. Adv. Manuf. Technol..

[16] Liu N., Lai Z., Cao Q., Huang Y., Chen M., Li C., Han X., Li L. (2020). Effects of the Inner/Outer Diameters of Flat Spiral Coils on Electromagnetic Sheet Metal Formation. Int. J. Adv. Manuf. Technol..

[17] Khan Z., Khan M., Jaffery S.H.I., Younas M., Afaq K.S., Khan M.A. (2020). Numerical and Experimental Investigation of the Effect of Process Parameters on Sheet Deformation during the Electromagnetic Forming of AA6061-T6 Alloy. Mech. Sci..

[18] Hu Q., Zhang F., Li X., Chen J. (2018). Overview on the Prediction Models for Sheet Metal Forming Failure: Necking and Ductile Fracture. Acta Mech. Solida Sin..

[19] Lou Y., Huh H., Lim S., Pack K. (2012). New Ductile Fracture Criterion for Prediction of Fracture Forming Limit Diagrams of Sheet Metals. Int. J. Solids Struct..

[20] COMSOL Failure Prediction in a Laminated Composite Shell. https://www.comsol.com/model/failure-prediction-in-a-laminated-composite-shell-65641.

[21] Mamalis A.G., Manolakos D.E., Kladas A.G., Koumoutsos A.K. (2004). Electromagnetic Forming and Powder Processing: Trends and Developments. Appl. Mech. Rev..

[22] Dond S.K., Kolge T., Choudhary H. Effect of Coil to Tubular Workpiece Magnetic Coupling on Electromagnetic Expansion Process. Proceedings of the 8th International Conference on High Speed Forming.

[23] Fenton G.K., Daehn G.S. (1998). Modeling of Electromagnetically Formed Sheet Metal. J. Mater. Process. Technol..

[24] Patil S.P., Prajapati K.G., Jenkouk V., Olivier H., Markert B. (2017). Experimental and Numerical Studies of Sheet Metal Forming with Damage Using Gas Detonation Process. Metals.

[25] Li F., Mo J., Zhou H., Fang Y. (2013). 3D Numerical Simulation Method of Electromagnetic Forming for Low Conductive Metals with a Driver. Int. J. Adv. Manuf. Technol..

[26] Liu W., Zhou H., Li J., Meng Z., Xu Z., Huang S. (2022). Comparison of Johnson-Cook and Cowper-Symonds Models for Aluminum Alloy Sheet by Inverse Identification Based on Electromagnetic Bulge. Int. J. Mater. Form..

[27] Hovorun T.P., Berladir K.V., Pererva V.I., Rudenko S.G., Martynov A.I. (2017). Modern Materials for Automotive Industry. J. Eng. Sci..

[28] Dordizadeh P., Gharghabi P., Niayesh K. (2011). Impact of Metal Thickness and Field Shaper on the Time-Varying Processes during Impulse Electromagnetic Forming in Tubular Geometries. J. Korean Phys. Soc..

[29] Paese E., Geier M., Homrich R.P., Rossi R., Rosa P. (2022). Assessing Experimental Apparatus for Sheet Metal Electromagnetic Forming Process Analysis. Mater. Manuf. Process..

[30] Kleiner M., Beerwald C., Homberg W. (2005). Analysis of Process Parameters and Forming Mechanisms within the Electromagnetic Forming Process. CIRP Ann. Manuf. Technol..

[31] Manes A., Peroni L., Scapin M., Giglio M. (2011). Analysis of Strain Rate Behavior of an Al 6061 T6 Alloy Selection and Peer-Review under Responsibility of ICM11. Procedia Eng..

[32] Woo M.A., Song W.J., Kang B.S., Kim J. (2019). Acquisition and Evaluation of Theoretical Forming Limit Diagram of al 6061-T6 in Electrohydraulic Forming Process. Metals.

[33] Nguyen H.H., Nguyen T.N., Nguyen T.N., Vu H.C. (2017). Forming limit curve determination of AA6061-T6 aluminum alloy sheet. Sci. Technol. Dev. J..

[34] Djavanroodi F., Derogar A. (2010). Experimental and Numerical Evaluation of Forming Limit Diagram for Ti6Al4V Titanium and Al6061-T6 Aluminum Alloys Sheets. Mater. Des..

[35] Golovashchenko S.F. (2007). Material Formability and Coil Design in Electromagnetic Forming. J. Mater. Eng. Perform..

[36] Psyk V., Kurka P., Kimme S., Werner M., Landgrebe D., Ebert A., Schwarzendahl M. (2015). Structuring by Electromagnetic Forming and by Forming with an Elastomer Punch as a Tool for Component Optimisation Regarding Mechanical Stiffness and Acoustic Performance. Manuf. Rev..

[37] Ouyang S., Li C., Du L., Li X., Lai Z., Peng T., Han X., Cao Q., Li L. (2021). Electromagnetic Forming of Aluminum Alloy Sheet Metal Utilizing a Low-Frequency Discharge: A New Method for Attractive Forming. J. Mater. Process. Technol..

[38] Xiong W., Wang W., Wan M., Li X. (2015). Geometric Issues in V-Bending Electromagnetic Forming Process of 2024-T3 Aluminum Alloy. J. Manuf. Process..

